# Cyclosporine A and IFNγ licencing enhances human mesenchymal stromal cell potency in a humanised mouse model of acute graft versus host disease

**DOI:** 10.1186/s13287-021-02309-6

**Published:** 2021-04-14

**Authors:** Jennifer M. Corbett, Ian Hawthorne, Hazel Dunbar, Ivan Coulter, Mairead Ni Chonghaile, Catherine M. Flynn, Karen English

**Affiliations:** 1 Cellular Immunology Laboratory, Department of Biology, Maynooth University, Maynooth, Co. Kildare Ireland; 2 Kathleen Lonsdale Institute for Human Health Research, Maynooth University, Maynooth, Co. Kildare, Ireland; 3 Sigmoid Pharma Ltd., Eden BioPharma Limited, NovaUCD, Belfield Innovation Park, University College Dublin, Dublin 4, Ireland; 4 Haematology department, St. James’s Hospital, Dublin, Ireland

**Keywords:** MSC cell therapy, Immunosuppressive drugs, Cyclosporine A, IFNγ, GvHD, SOCS1

## Abstract

Immunosuppressive ability in human MSC donors has been shown to be variable and may be a limiting factor in MSC therapeutic efficacy in vivo. The importance of cytokine activation of mesenchymal stromal cells (MSCs) to facilitate their immunosuppressive function is well established. This study sought to further understand the interactions between MSCs and the commonly used calcineurin inhibitor cyclosporine A (CsA). The existing literature regarding approaches that use MSCs and cyclosporine are conflicting regarding the effect of CsA on MSC potency and function. Here, we clearly demonstrate that when added at the same time as MSCs, CsA negatively affects MSC suppression of T cell proliferation. However, licencing MSCs with IFNγ before addition of CsA protects MSCs from this negative effect. Notably, adding CsA to MSCs after IFNγ pre-stimulation enhances MSC production of IDO. Mechanistically, we identified that CsA reduces SOCS1 expression to facilitate enhanced IDO production in IFNγ pre-stimulated MSCs. Importantly, CsA exposure to IFNγ pre-stimulated MSC before administration, significantly enhanced the potency of MSCs in a human relevant humanised mouse model of acute Graft versus Host Disease. In summary, this study identified a novel licencing strategy to enhance MSC potency in vitro and in vivo.

**Supplementary Information:**

The online version contains supplementary material available at 10.1186/s13287-021-02309-6.

## Introduction

Clinical studies have demonstrated the benefits of MSCs as a cell therapy, particularly in acute Graft versus Host Disease (aGvHD), solid organ transplantation and for the treatment of complex perianal fistulas in Crohn’s disease patients [1–4]. In the context of GvHD, variability in patient response to MSC therapy has been a major hurdle in the translation of MSC therapy for routine clinical application [5, 6]. In vivo studies from our groups and others have clearly shown that MSCs require a threshold level of pro-inflammatory cytokines to exert immunosuppressive effects in aGvHD [7, 8]. We and others have demonstrated the important role for endogenous or pro-inflammatory cytokine licencing of MSCs in an array of inflammatory disease models including aGvHD [8, 9].

In vivo, MSCs respond to inflammation and adopt immunoregulatory mechanisms accordingly. Given that MSCs may be exposed to immunosuppressive drugs following administration in patients. We sought initially to understand the effect that immunosuppressive (IS) drugs have on the activation status and subsequent function of MSCs.

Cyclosporine (CsA) is an immunosuppressive drug commonly used to prevent rejection of transplanted organs, to treat autoimmune disorders and for prophylaxis or treatment of GvHD [10, 11]. In these settings, T cells are the central mediators associated with initiating and maintaining these unwanted inflammatory responses. CsA targets T cells by preventing the transcription of cytokine genes fundamental for T cell proliferation, including IL2, by hindering calcium-dependent signal transduction pathways [12, 13]. However, little is known about the direct interaction of CsA with MSCs in terms of the impact CsA may have on MSC immunosuppressive capacity.

Buron et al. briefly assessed the interactions of MSCs with IS drugs in vitro using proliferation assays and revealed that combination of MSCs with CsA or rapamycin antagonised the suppression of alloantigen-driven PBMC proliferation [14]. In other studies rapamycin [15] and budesonide [16] enhanced the potency of MSC immunosuppression in vitro and in vivo mediated by the pre-treated uptake of the drugs within the cell as opposed to co-addition of MSCs with the drugs.

Suppressor of cytokine signalling 1 (SOCS1) is a negative regulator of cytokine signal transduction and plays an important role in regulating IFNγ signalling [17]. SOCS1 has recently been described as being a negative regulator of MSC immunosuppressive ability by reducing the expression of inducible nitric oxide synthase (iNOS) [18]. CsA is known to interfere with the inhibitory function of SOCS1 in cells infected with hepatitis C virus or rotavirus [19, 20]. Therefore, it was important to investigate the effect CsA has on SOCS1 signalling in MSC.

This study shows that cyclosporine reduced MSC production of IDO and suppression of T cell proliferation in vitro. Importantly, this study identified that pre-stimulation of MSCs with IFNγ 24 h before exposure to CsA enhances MSC immunomodulatory capacity in vitro and in vivo in a humanised mouse model of acute GvHD. Mechanistically, this effect may be associated with CsA-driven downregulation of SOCS1 in MSCs. This study has identified a novel means to enhance MSC potency which may lead to enhanced MSC therapeutic efficacy in the clinic.

## Materials and methods

### Ethical approval

All procedures involving the use of animals or human materials were carried out by licenced personnel. Ethical approval for all work was granted by the biological research ethics committee of Maynooth University (BRESC-2013-13) and by the research ethics committee at St. James’s Hospital (2012/11/05). Human bone marrow aspirate was harvested from healthy adult donors under informed consent.

### Culture of human mesenchymal stromal cells (MSC)

Bone marrow-derived mesenchymal stromal cells were generated by collaborators at NUI Galway or at Maynooth University using donated bone marrow aspirates from St. James’s hospital. Briefly, bone marrow aspirates were taken from the iliac crest of donor patients according to an approved clinical protocol [21]. Isolated human MSC were resuspended in complete DMEM (Sigma-Aldrich, Wicklow, Ireland) supplemented with 10% (v/v) fetal bovine serum (FBS) (Labtech, Uckfield, UK), 50 U/ml penicillin (Sigma-Aldrich) and 50 μg/ml streptomycin (Sigma-Aldrich) and cultured at 37 °C in 5% C0_2_. MSC were used in experiments between passages 2–6.

### T cell suppression assay

Human PBMCs were isolated from buffy packs (Irish Blood Transfusion Service), by Ficoll density gradient centrifugation. 5 × 10^4^ Carboxyfluorescein succinimidyl ester (CFSE) labelled PBMC were co-cultured (Fisher, Ballycoolin, Ireland) with MSC (1 × 10^4^/well) (1:5). In the presence of CD3/CD28 Dynabeads® beads (Gibco) (1 × 10^4^/well). After 4 days, PBMC were harvested and the level of proliferation by CD3^+^ cells was analysed by flow cytometry (Accuri C6, BD Biosciences) and enumerated using counting beads (3 × 10^5^/ml) (Calibrite™ Beads, BD Biosciences). To examine MSC production of IDO following co-culture with activated PBMC, MSC were labelled with cell proliferation dye eFluor® 670. For analysis of intracellular IDO, cells were incubated with 1X Brefeldin A (eBioscience) for the last 4 h of the co-culture on the third day of the PBMC suppressor assay followed by preparation using the intracellular FoxP3 kit (eBioscience) as per manufacturer’s instructions and incubation with IDO PE antibody (eBioscience) for 45 min. Cells were then washed in FACs buffer and acquired using the Accuri C6 gating on the cell proliferation dye eFluor® 670 labelled human MSC.

### Stimulation of human MSC with pro-inflammatory cytokines and cyclosporine a

Human MSC (2 × 10^5^/well) were allowed to adhere overnight in a 6 well tissue culture plate. MSC were stimulated with rhIFNγ (Peprotech, London, UK) (50 ng/ml) and/or Cyclosporine A (Sigma-Aldrich) at 100, 500, or 1000 ng/ml for up to 24 h. Importantly, for some experiments, MSC was stimulated with rhIFNγ for 24 h followed by CsA for 24 h.

### Real-time PCR

cDNA was analysed for the quantification of mRNA expression. Briefly, cDNA (1 μg) was amplified in the presence of SYBR® Green JumpStart™ Taq ReadyMix (Sigma-Aldrich).

Accumulation of gene-specific products was measured continuously by means of fluorescence detection over 40 cycles. Expression was quantified in relation to the housekeeper GAPDH using the ∆CT method. The fold change in relative gene expression was determined by calculating the 2^-∆CT^ values. The following PCR predesigned primers (Sigma Aldrich) were used: GAPDH: 5′-3′ ACAGTTGCCATGTAGACC and 3′-5′ TTTTTGGTTGAGCACAGG, CCL2: 5′-3′ AGACTAACCCAGAAACATCC and 3′-5′ ATTGATTGCATCTGGCTG, CXCL9: 5′-3′ AGGTCAGCCAAAAGAAAAAG and 3′-5′ TGAAGTGGTCTCTTATGTAGTC, COX2: 5′-3′ AAGCAGGCTAATACTGATAGG and 3′-5′ TGTTGAAAAGTAGTTCTGGG and IDO: 5′-3′ TTGTTCTCATTTCGTGATGG and3’-5′ TACTTTGATTGCAGAAGCAG.

### Elisa

ELISAs (IFNγ, TNFα, IL-2 or CXCL9) were carried out according to manufacturer’s instructions (R & D Systems, Abingdon, UK or eBioscience, Paisley, Scotland).

### Western blotting for IDO, SOCS1, pSTAT1, STAT1 and actin

Protein extraction: Intracellular protein was extracted from adherent MSC. Cell pellets were then resuspended in 90 μl RIPA lysis buffer. Protein lysates were subjected to centrifugation at 12,000 g for 10 min at 4 °C. 90 μl of the supernatant which constitutes the intracellular protein was added to a1.5ml tube and stored at − 20 °C. Prior to loading protein lysates, samples were mixed with 4X Laemmli sample buffer and boiled for 5 min.

SDS-PAGE was carried out in accordance with the Laemmli method as modified by Studier. Samples and appropriate prestained (10–180 kDa) protein markers were loaded into separate 0.75 mm wells. Electrophoresis was performed at 60 V through a 5% polyacrylamide stacking gel and then through a 8–15% polyacrylamide resolving gel at 80 V for up to 2 h.

Immunoblotting: Proteins were transferred electrophoretically to nitrocellulose membranes (GE Healthcare, Buckinghamshire, England) in a Hoefer TE 70 Semiphor semi-dry transfer unit (GE Healthcare) at 100 mA for between 40 and 80 min depending on protein size. Following transfer, non-specific binding was blocked with blocking buffer (tris-buffered saline (TBS)), 0.1% (v/v) Tween-20 with 5% (w/v) non-fat dry milk) under gentle agitation. The membranes were then incubated under agitation at 4 °C overnight with the primary antibodies (IDO (D5J4E) 1:1000 dilution, SOCS1 (A156) 1:500 dilution, Stat1 (D1K9Y) 1:1000 dilution, pStat-1 (Y701) 1:1000 dilution, beta-Actin (8H10D10) 1:5000 dilution, all from Cell Signalling) diluted in TBS containing 0.1% (v/v) Tween-20 (TBST) with 5% (w/v) skimmed milk powder or BSA. The membranes were subsequently subjected to 3 × 5 min washes in TBST. Membranes were then incubated in a secondary antibody (anti-mouse HRP 1:1000 dilution or anti-rabbit HRP 1:1500 dilution both from Cell Signalling) in TBST containing 5% (w/v) skimmed milk powder for 1 h in the dark at room temperature. The membranes were then washed a further 3 times for 5 min each in TBST in the dark. The immunoreactive bands were detected using enhanced chemiluminescence development (WesternBright ECL HRP Substrate, Advansta, Labtech).

### Kynurenine assay to assess indoleamine 2,3-dioxygenase activity

60ul of MSC cell culture supernatant was mixed with 30ul of 30% trichloroacetic acid (Merck) and incubated for 30 min at 50 °C followed by centrifugation at 3000*g* for 5 min. 75ul of the supernatant was transferred to a 96 well flat bottomed plate followed by the addition of 75ul of freshly prepared Ehrlich reagent (Sigma Aldrich, 2% p-dimethylaminobenzaldehyde in glacial acetic acid (Merck)) for 12–30 min. The absorbance was read at 492 nm in a microplate reader. The concentration of Kynurenine was calculated using a standard curve of L-Kynurenine (Sigma-Aldrich) (0–1250uM).

### Humanised mouse model of acute graft versus host disease

As previously described [8], NOD.Cg-Prkdc^scid^IL2^tmlWjl^/Szj (NOD-Scid IL-2rγnull) (NSG) were exposed to a conditioning dose of 2.4 Grey (Gy) whole body gamma irradiation. Freshly isolated human peripheral blood mononuclear cells (PBMCs) (8.0 × 10^5^/g) (buffy coat packs were supplied by the Irish Blood Transfusion Service) were administered by intravenous injection to the tail vein using a 27 gauge needle and a 1 ml syringe 4–8 h following irradiation. Human bone-marrow derived MSC (6.4 × 10^4^/gram) [MSC], IFNγ pre-stimulated (24 h) MSC [MSCγ], CsA pre-stimulated (24 h) MSC [MSC CsA] or MSC that were IFNγ pre-stimulated (24 h) followed by CsA pre-stimulation (24 h) [MSCγ CsA] were administered intravenously on day 6. *N* = 9 mice per group. The mice were scored daily at disease onset to monitor animal welfare and disease progression and the percentage survival recorded out to 30 days.

### Statistical analysis

The students unpaired *t* test was used when statistical analysis was required between two experimental groups. One way ANOVA Multiple Tukey comparison test was used to test for statistical significance of differences when multiple experimental groups were compared. Mantel-Cox test (log rank test) was used to compare survival between treatment groups. The ratio for median survival was computed using GraphPad Prism.

## Results

### IFNγ licencing safeguards MSCs from negative effects of CsA co-treatment

**Fig. 1:**
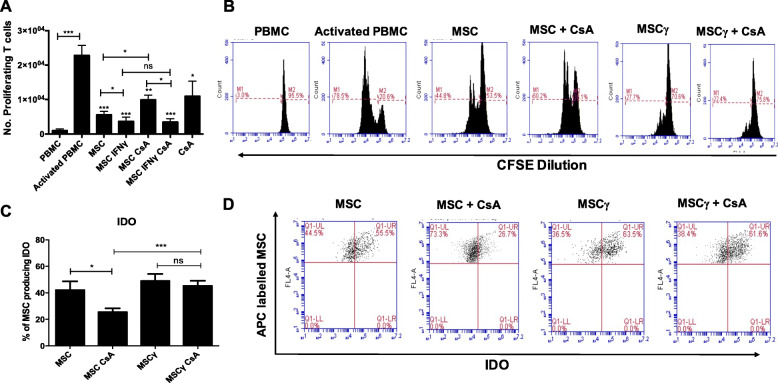
IFNγ licencing safeguards MSC from negative effects of CsA co-treatment. MSCs were seeded (1 × 10^4^ per well) into a 96 well round bottom plate followed by the addition of anti-CD3/CD28 bead (1 × 10^4^ per well) activated CFSE labelled PBMCs (5 × 10^4^ per well). MSC IFNγ and MSC IFNγ CsA groups were pre-stimulated with IFNγ (50 ng/ml) for 24 h. Some groups were cultured in the presence of CsA (1000 ng/ml). On day four, cells were harvested and stained with anti-CD3 and 7AAD viability dye to analyse CD3^+^ proliferation by flow cytometry. CsA significantly antagonises the immunosuppressive ability of MSC, *n* = 7 (**a**) and representative histogram plots (**b**). MSC alone or stimulated with IFNγ (50 ng/ml) for 24 h were labelled with eFluor® 670 and co-cultured with anti-CD3/28 driven PBMCs. Labelled MSC were harvested (on day 3), fixed, permeabilised and stained intracellularly with fluorescent antibody for IDO and analysed by flow cytometry Data presented as mean +/− SEM of the % of MSCs producing IDO (*n* = 5) (**c**) and representative histograms (**d**). Statistical analysis was carried out using one way ANOVA Multiple Tukey comparison test and unpaired Student’s *t* test where *< 0.05, **< 0.01 and *** < 0.001. Stars with no bar are in comparison to the activated PBMC group. ns not significant

CsA negatively impacts MSC capacity to suppress T cell proliferation in vitro in a dose-dependent manner (Supplementary Figure 1). Notably there was a significant difference between the suppression mediated by MSCs and MSCs in the presence of high concentrations of CsA (MSC CsA) (Fig. 1a, b). Importantly, the presence of CsA did not impair T cell suppression mediated by IFNγ pre-licenced MSCs (Fig. 1a, b). The influence of IFNγ and CsA on IDO-mediated immunosuppression by MSCs was examined further using MSC labelled with the cell proliferation dye eFluor® 670 in a CFSE-labelled PBMC proliferation co-culture assay. The percentage of MSCs and IFNγ licenced MSCs producing IDO were similar on day 4 in the activated PBMC co-culture (Fig. 1c), as there is likely plenty of IFNγ present to activate MSC production of IDO. CsA was shown to have a significant negative effect on the percentage of MSCs producing IDO (Fig. 1c) correlating with reduced suppression levels (Fig. 1a, b). In the presence of CsA, the percentage of IFNγ licenced MSCs (MSCγ CsA) producing IDO is significantly higher (*P* < 0.001) than MSCs with CsA (MSC CsA). Taken together, these data suggest that IFNγ licencing of MSCs facilitates a beneficial immunosuppressive effect in combination with CsA.

### CsA enhancement of IDO production by IFNγ-activated MSCs is associated with a downregulation of SOCS1

**Fig. 2:**
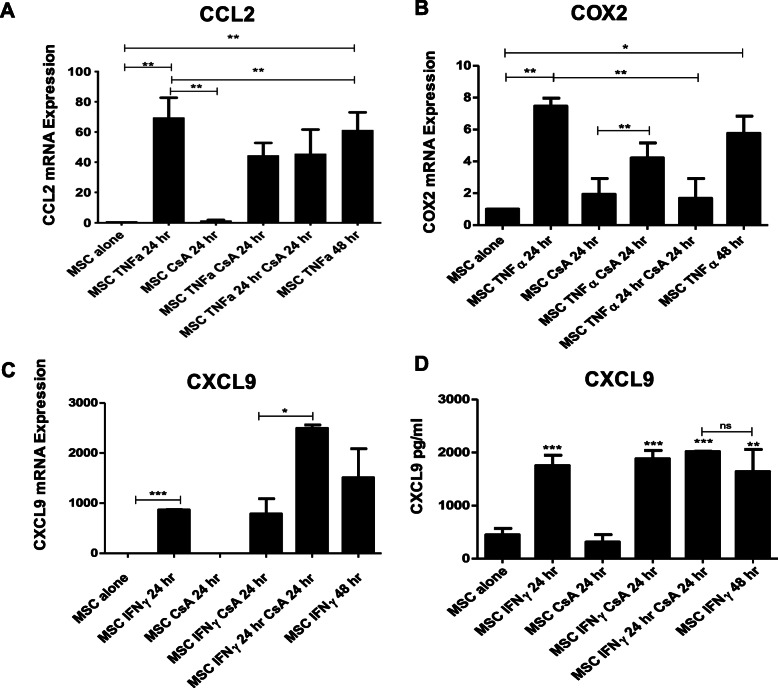
CsA enhances MSC immunomodulatory gene expression induced by IFNγ but not TNFα. MSCs were seeded at 2 × 10^5^ per well in a 6 well plate and left alone or stimulated with IFNγ (50 ng/ml) or TNFα (20 ng/ml) and/or CsA (1000 ng/ml) at different timepoints. In some groups, MSCs were first stimulated with one cytokine for 24 h followed by CsA for a further 24 h. After a total of 48 h, supernatant was collected for detection of CXCL9 by ELISA and cells were harvested for Quantitative PCR (qPCR) analysis. In other groups, MSCs were stimulated with a cytokine and CsA and harvested after 24 h. Controls for exposure of cytokines at both 24 h and 48 h timepoints were performed. RNA from each sample was extracted, cDNA was synthesised and used as a template for qPCR. mRNA expression of CCL2 (**a**), COX2 (**b**) and CXCL9 (**c**) was calculated relative to the house keeping gene GAPDH. The concentration of CXCL9 was examined by ELISA (**d**). These experiments were repeated using four MSC donors (*n* = 4) (passage 5–7), and the results shown are representative of this. Statistical analysis was carried out using unpaired Student’s *t* test where * < 0.05, ** < 0.01 and *** < 0.001. Stars with no bar are in comparison to the MSC alone group

**Fig. 3:**
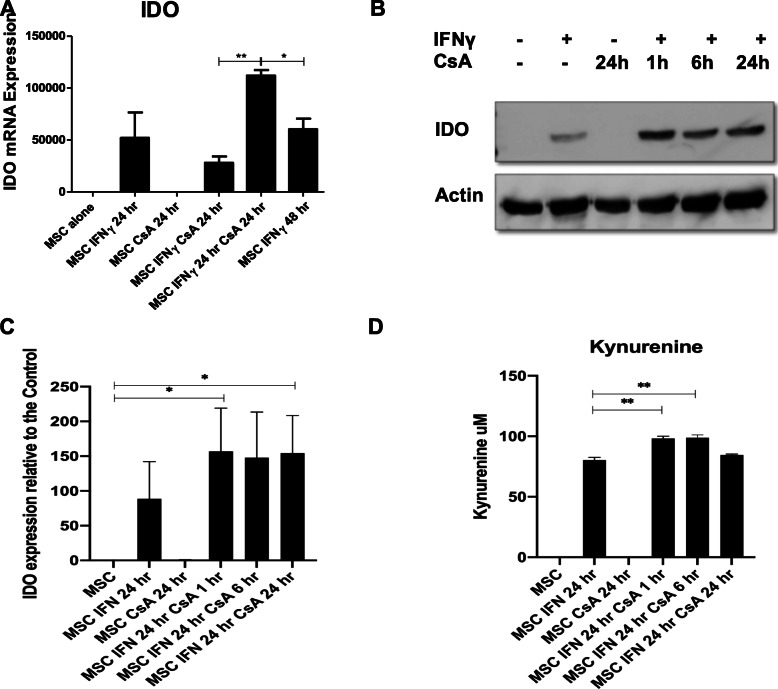
CsA enhances IDO production and activity by IFNγ activated MSC. MSCs were seeded at 2 × 10^5^ per well in a 6 well plate and left alone or stimulated with IFNγ (50 ng/ml) and/or CsA (1000 ng/ml) at different timepoints. In some groups, MSCs were first stimulated with IFNγ for 24 h followed by CsA for a further 24 h. After a total of 48 h, cells were harvested for Quantitative PCR (qPCR) analysis. In other groups, MSCs were stimulated with IFNγ and CsA and harvested after 24 h. Controls for exposure of cytokines at both 24 h and 48 h timepoints were performed. RNA from each sample was extracted, cDNA was synthesised and used as a template for qPCR. mRNA expression of IDO (**a**) was calculated relative to the house keeping gene GAPDH. The concentration of kynurenine was examined by colorimetric assay using a L-kynurenine standard curve to calculate kynurenine concentration (**d**). These experiments were repeated using four MSC donors (*n* = 4) (passage 5–7) and the results shown are representative of this. Statistical analysis was carried out using unpaired Student’s *t* test where * < 0.05, ** < 0.01 and *** < 0.001. Stars with no bar are in comparison to the MSC alone group

To investigate the potential for CsA to enhance MSC immunosuppression, in addition to IDO, a number of genes known to play a role in MSC immunomodulatory capacity were examined. As both TNFα and IFNγ stimulated genes have been implicated in MSC immunomodulation, immunomodulatory genes driven by these cytokines were examined. CCL2 (Fig. 2a) and COX2 (Fig. 2b) mRNA were both significantly upregulated by TNFα in MSCs, and addition of CSA 24 h after TNFα pre-stimulation (MSC TNFα 24 h CsA 24 h) significantly reduced COX2 mRNA expression. Differentially, mRNA expression levels of CXCL9 (Fig. 2c) and IDO (Fig. 3a) were significantly increased in MSCs stimulated with IFNγ followed by CsA 24 h later (MSC IFNγ 24 h CsA 24 h). At the protein level, there was no increase in CXCL9 (Fig. 2d) above that in MSCs stimulated with IFNγ. The tryptophan depleting enzyme IDO is regulated by IFNγ and has been shown to be altered in human MSCs following treatment with steroids [16, 22, 23]. Although IDO protein was not induced by CsA alone, the IFNγ induction (24 h stimulation) of IDO production by licenced MSC was enhanced by the presence of CsA at 1 h, 6 h and 24 h timepoints following the addition of IFNγ in MSCs (Fig. 3b, c). Moreover, IDO activity measured using a kynurenine assay was significantly increased by the presence of CsA at 1 h and 6 h following IFNγ stimulation (Fig. 3d). This suggests that while IDO expression, production and activity increases upon exposure to IFNγ, additional conditioning by CsA resulted in a further increase of IDO production and activity. This effect was specific to the IFNγ regulated gene IDO and was not the case for the TNFα regulated gene COX-2.

**Fig. 4:**
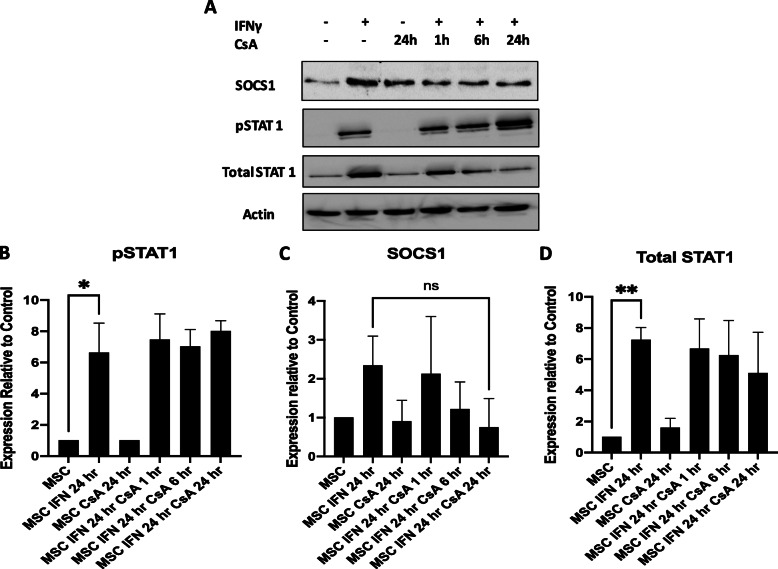
Effects of CsA on IFNγ signalling pathway proteins SOCS1 and pSTAT1. Protein levels of SOCS1, pSTAT1 and total STAT1 were measured by western blot in MSC stimulated with/out IFNγ (50 ng/ml) for at least 24 h followed by exposure to CsA (1000 ng/ml) for 1 h, 6 h or 24 h for the remainder of a 48 h window. All cells were harvested at a 48 h time-point. β-Actin was included as a loading control. Densitometry of *n* = 3 studies is shown for SOCS1 (**b**), pSTAT1 (**c**) and total STAT1 (**d**). Statistical analysis was carried out using unpaired Student’s *t* test where * < 0.05 and ** < 0.01

SOCS1 has been described as a regulator of MSC immunosuppressive functions through IFNγ signalling. MSCs were licenced with IFNγ, stimulated with CsA for 1 h, 6 h or 24 h and the protein levels of SOCS1, pSTAT1 and STAT1 were analysed by Western blot to assess the effect CsA has on this signalling pathway. CsA downregulated SOCS1 protein in IFNγ licenced MSCs in a time dependent manner, most notably at 6 h and 24 h (Fig. 4a, b). pSTAT1 protein levels were significantly increased in IFNγ licenced MSCs, but CsA has no significant impact on pSTAT1 protein expression (Fig. 4a, c). IFNγ also upregulated expression of total STAT1 in MSCs and CsA had no impact on total STAT1 (Fig. 4d). Interestingly, CsA alone did not downregulate SOCS1 protein in MSCs (Fig. 4a, b), suggesting that SOCS1 inhibition of CsA is transient, indirect and time dependent on IFNγ activation of MSCs.

### Pre-licencing of MSCs with IFNγ followed by CsA promotes MSCs efficacy in prolonging survival in a humanised mouse model of acute GvHD

**Fig. 5:**
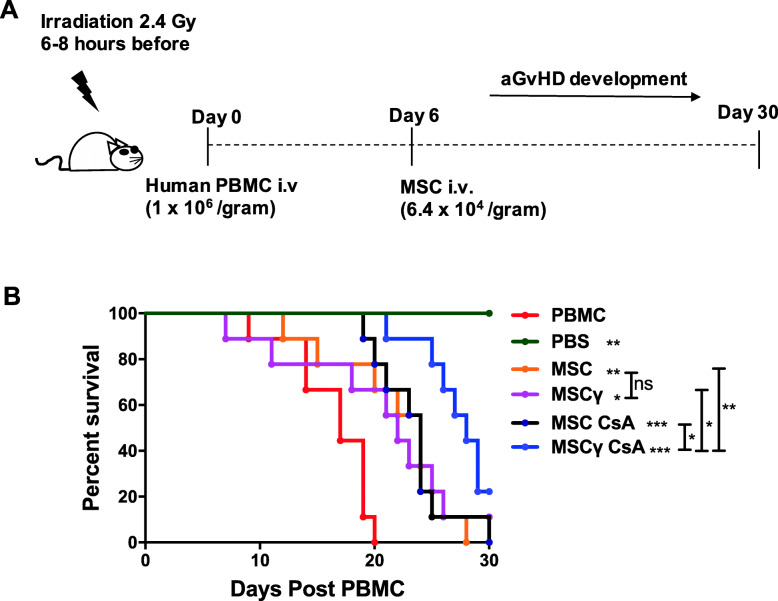
Prestimulation with CsA enhances the potency of IFNγ licenced MSC in a humanised mouse model of acute GvHD. A humanised mouse model of aGvHD was established by administering 8 × 10^5^ human PBMC/gram (or PBS as a control) to irradiated (2.4 Gy) NSG mice on day 0. 6.4 × 10^4^/g MSC, IFNγ (50 ng/ml 24 h) licenced MSC [MSCγ], MSC cultured with CsA (1000 ng/ml) for 24 h [MSC CsA] or MSC stimulated with IFNγ (50 ng/ml) for 24 h followed by CsA (1000 ng/ml) for 24 h [MSCγ CsA] were delivered intravenously on day 6. Mice were monitored for aGvHD development every 2 days until day 9 and then every day for the duration of the experiment (**a**). Survival curve (**b**). *n* = 9 mice per group. Statistical analysis was carried out using a Mantel-Cox test for the survival curve where * < 0.05, ** < 0.01 and *** < 0.001. * with no bar are in comparison to the PBMC group

Here, we used a human relevant system to test the hypothesis that pre-licencing of MSCs with IFNγ followed by CSA enhances MSC potency in suppressing T cell-driven inflammatory disease. In particular, we explored the enhancing effect of CsA on IFNγ pre-licenced MSC capacity to suppress pathogenic T cell responses and prolong survival in an humanised mouse model of acute graft versus host disease (Fig. 5a). Previously we have demonstrated that MSCs administered on day 6/7 significantly prolong survival and that while IFNγ pre-licenced MSCs have similar efficacy to unstimulated MSCs at day 6/7, this option is therapeutically superior to conventional MSCs when administered on day 0 [8]. Administration of a single dose of human MSCs or IFNγ activated MSCs (MSCγ) on day 6 also significantly prolongs survival at day 30 (Fig. 5b). Administration of MSCs pre-exposed to CsA for 24 h (MSC CsA) similarly prolonged survival to that of the MSC and the MSCγ groups. Importantly, MSCγ CsA (IFNγ activated MSC for 24 h followed by exposure to CsA for 24 h) prolonged survival in the aGvHD mice with significantly greater potency than any of the other groups. This study suggests that the efficacy of MSCs can be enhanced by exposure to CsA in MSCs previously licenced with IFNγ and indicates that human trials of MSCs licenced in this novel way could be worthwhile.

## Discussion

The ability of MSCs to suppress the adaptive immune system is a key feature that underlies both the mechanism of action as well as interest in the development of MSC therapies. In particular, MSCs have been shown to have dynamic interactions with T cells via cell contact or through secreted soluble factors in the context of specific environmental cues [24–27]. MSCs suppress T cell proliferation in a dose dependent manner. Importantly, a recent study has shown that inhibiting protein transport in PBMCs impairs MSC immunosppressive capacity in co-cultures. Moreover, a 4-fold reduction in PBMC number reduced MSC efficacy in an MSC-PBMC suppressor assay [28], highlighting that MSCs require a threshold level of activation by pro-inflammatory cytokines like TNFα and IFNγ [29]. CsA also targets T cells and is a potent T cell immunosuppressive drug that suppresses the production of IL2 and other pro-inflammatory cytokines (including TNFα and IFNγ) as a result of calcineurin inhibition [12, 13].

Here we showed that CsA has an inhibitory effect on MSC immunosuppressive ability. Similarly others showed that CsA anatagonised the suppressive effects of MSCs [14]. Others observed that CsA only added slightly to the inhibitory effect of MSCs [30] while Maccario et al. [31] showed that CsA and MSCs combined had synergistic suppressive effects in a mixed lymphocyte culture. Notably, in that study CsA was added at 50 ng/ml whereas in our study 1000 ng/ml CsA was added which might explain these differences.

We have shown that CsA significantly enhanced IDO production by pre-licenced MSCs at the protein level. Importantly, when CsA alone was added to MSCs it did not induce IDO production; but, when CsA was added after IFNγ licencing of MSCs, IDO production and activity was enhanced. These findings support the finding by Ankrum et al. [16] that IDO was shown to be enhanced in human MSCs following treatment with IFNγ and subsequent steroids, budesonide and dexamethasone. It is important to distinguish that the data presented here are due to direct effects of CsA on MSC production of IDO following IFNγ pre-stimulation rather than effects observed in a PBMC co-culture assay. In this way, the stimulus for facilitating this effect was solely down to IFNγ and that the IFNγ (but not TNFα) pre-licencing of MSCs was identified as being key for facilitating this effect.

The next approach was to probe this pathway further and identify a mechanism by which this effect was being achieved in vitro*.* Although not significant, there was a trend whereby CsA had a time dependent inhibitory effect on SOCS1 expression at the protein level in MSCs which may lead to the preservation of the IFNγ pathway. The trend of a reduction in SOCS1 mediated by CsA was found only in IFNγ licenced MSC as CsA alone had no inhibitory effect on basal levels of SOCS1 in resting MSCs which further supports the requirement for IFNγ to induce IDO production. These findings might suggest that exploitation of SOCS1 inhibition by CsA could improve MSC therapy by enhancing their potency via an IDO-mediated mechanism.

We have shown conclusively for the first time that licencing of MSCs is required for MSC to retain their suppressive function in the presence of CsA in vitro. We have translated these in vitro findings to a human relevant condition, demonstrating that CsA can be used to enhance the potency of IFNγ licenced MSC in a humanised mouse model of acute GvHD. We have also identified that CsA can enhance IDO production in IFNγ-licenced MSC and provide some new data which suggests that CsA may have an inhibitory effect on SOCS1 in IFNγ-licenced MSC. A limitation of our study is that it does not show that a downregulation of SOCS1 is definitively associated with the enhanced IDO production and MSC immunomodulatory potency. Importantly, support is provided by studies demonstrating that SOCS1 knockdown in MSCs leads to enhanced immunosuppressive effects with increased nitric oxide and PGE-2 following stimulation with proinflammatory cytokines [18, 32]. Nonetheless, this study identifies a novel role for CsA in maintaining signal transduction in the IFNγ pathway of MSC possibly through the inhibition of SOCS1 which has consequences for the potency of MSC immunosuppressive function. Importantly, these findings represent an advance in our understanding of how CsA interacts with MSCs, particularly identifying MSC activation and timing of CsA as being crucial for beneficial immunosuppressive functions which can be applied to in vivo pre-clinical models and clinical settings.

## Supplementary Information

**Additional file 1: Supplementary Figure 1.** CsA impairs MSC suppression of T cell proliferation in vitro. MSC were seeded (1 × 10^4^ per well) into a 96 well round bottom plate followed by the addition of anti-CD3/CD28 bead (1 × 10^4^ per well) activated CFSE labelled PBMCs (5 × 10^4^ per well). Some groups were cultured in the presence of CsA (100, 500 or 1000 ng/ml). On day four, cells were harvested and stained with anti-CD3 and 7AAD viability dye to analyse CD3^+^ proliferation by flow cytometry. Statistical analysis was carried out using one way ANOVA Multiple Tukey comparison test and unpaired student *t*-test where * < 0.05, ** < 0.01 and *** < 0.001. Stars with no bar are in comparison to the activated PBMC group.

## Data Availability

The datasets during and/or analysed during the current study available from the corresponding author on reasonable request.
