# Supplementary figures and images for: Cyclosporine A and IFNγ licencing enhances human mesenchymal stromal cell potency in a humanised mouse model of acute graft versus host disease
Source: Stem Cell Res Ther. 2021 Apr 14;12:238. doi: 10.1186/s13287-021-02309-6 (PMC8048195; doi:10.1186/s13287-021-02309-6)

Supplementary Figure 1

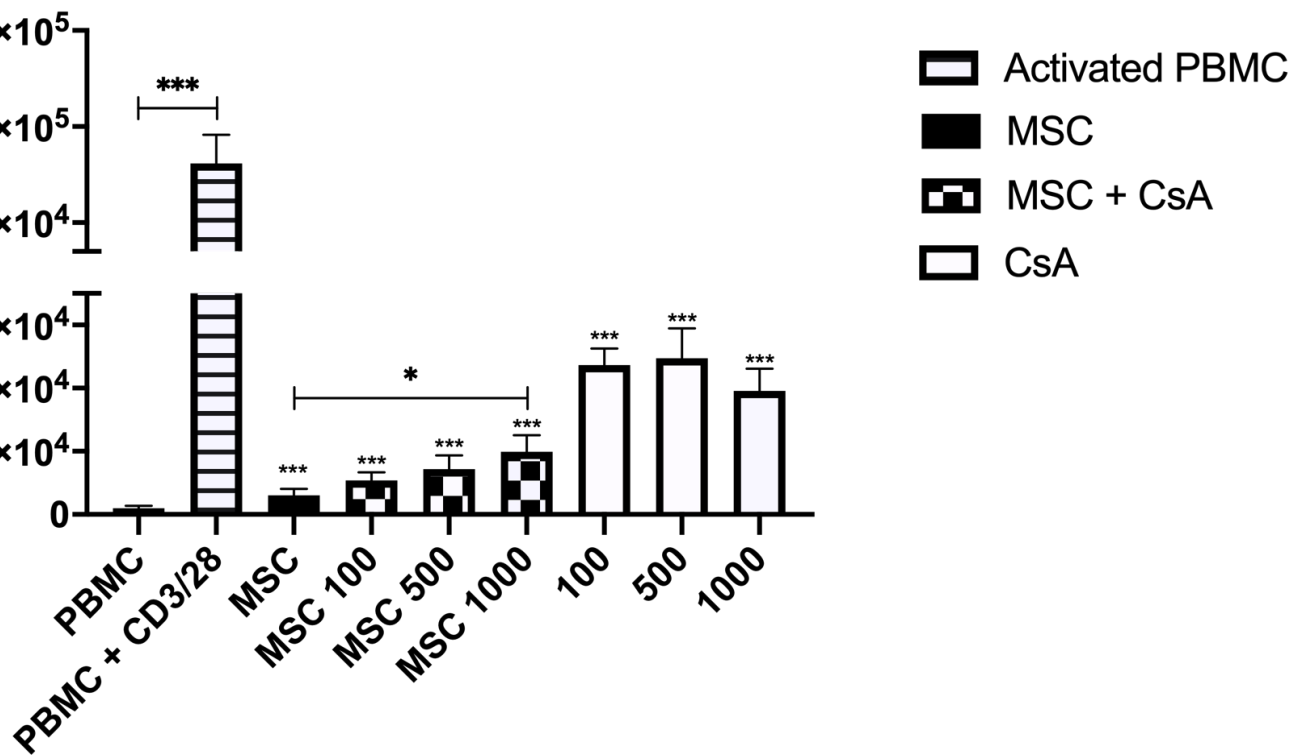

Supplement: Supplementary file 1 — Additional file 1: Supplementary Figure 1. CsA impairs MSC suppression of T cell proliferation in vitro. MSC were seeded (1 × 10^4^ per well) into a 96 well round bottom plate followed by the addition of anti-CD3/CD28 bead (1 × 10^4^ per well) activated CFSE labelled PBMCs (5 × 10^4^ per well). Some groups were cultured in the presence of CsA (100, 500 or 1000 ng/ml). On day four, cells were harvested and stained with anti-CD3 and 7AAD viability dye to analyse CD3+ proliferation by flow cytometry. Statistical analysis was carried out using one way ANOVA Multiple Tukey comparison test and unpaired student t-test where * < 0.05, ** < 0.01 and *** < 0.001. Stars with no bar are in comparison to the activated PBMC group. [file 13287_2021_2309_MOESM1_ESM.pdf]
